# Evaluating the effectiveness of a multifaceted intervention to reduce low-value care in adults hospitalized following trauma: a protocol for a pragmatic cluster randomized controlled trial

**DOI:** 10.1186/s13012-023-01279-y

**Published:** 2023-07-07

**Authors:** Lynne Moore, Mélanie Bérubé, Amina Belcaid, Alexis F. Turgeon, Monica Taljaard, Robert Fowler, Natalie Yanchar, Éric Mercier, Jérôme Paquet, Henry Thomas Stelfox, Patrick Archambault, Simon Berthelot, Jason R. Guertin, Barbara Haas, Noah Ivers, Jeremy Grimshaw, Alexandra Lapierre, Yongdong Ouyang, Michael Sykes, Holly Witteman, Paule Lessard-Bonaventure, Belinda Gabbe, François Lauzier, The Canadian Traumatic Brain Injury Research Consortium (CTRC)

**Affiliations:** 1 Department of Social and Preventive Medicine, Université Laval, 1050 Av. de La Médecine, Québec, Qc Canada; 2 Population Health and Optimal Health Practices Research Unit, Centre de Recherche du CHU de Québec (Hôpital de L’Enfant-Jésus), Université Laval, 1050 Av. de La Médecine, Québec, Qc Canada; 3 Faculty of Nursing, Université Laval, 1050 Av. de La Médecine, Québec, Qc Canada; 4 Institut national d’excellence en santé et services sociaux, Bd Laurier, Québec, Qc 2535 Canada; 5 Department of Anesthesiology and Critical Care Medicine, Division of Critical Care Medicine, Université Laval, 1050 Av. de La Médecine, Québec, Qc Canada; 6 Ottawa Hospital Research Institute, 725 Parkdale Ave, Ottawa, On Canada; 7 Sunnybrook Research Institute, 2075 Bayview Avenue, Toronto, On Canada; 8 Department of Surgery, University of Calgary, 3280 Hospital Dr. NW, Calgary, Ab Canada; 9 Department of Surgery, Université Laval, 1050 Av. de La Médecine, Québec, Qc Canada; 10 Department of Critical Care Medicine, Medicine and Community Health Sciences, O’Brien Institute for Public Health, University of Calgary, 3280 Hospital Dr. NW, Calgary, Al Canada; 11 Department of Surgery, University of Toronto, 149 College St, Toronto, On Canada; 12 Department of Family and Community Medicine and Institute of Health Policy, Management and Evaluation, University of Toronto, 155 College St 4Th Floor, Toronto, On Canada; 13 Faculty of Nursing, Université de Montréal, Chem. de La Côte-Sainte-Catherine, Montréal, Qc 2375 Canada; 14 Department of Nursing, Midwifery, and Health, Northumbria University, Ellison PI, Newcastle, UK; 15 Department of Family and Emergency Medicine, Université Laval, 1050 Av. de La Médecine, Québec, Qc Canada; 16 Department of Surgery, Division of Neurosurgery, Université Laval, 1050 Av. de La Médecine, Québec, Canada; 17 School of Public Health and Preventive Medicine, Monash University, 553 St. Kilda Rd, Melbourne, Victoria VIC 3004 Australia

**Keywords:** Low-value practice, Trauma system, Multifaceted intervention, Cluster randomized controlled trial

## Abstract

**Background:**

While simple Audit & Feedback (A&F) has shown modest effectiveness in reducing low-value care, there is a knowledge gap on the effectiveness of multifaceted interventions to support de-implementation efforts. Given the need to make rapid decisions in a context of multiple diagnostic and therapeutic options, trauma is a high-risk setting for low-value care. Furthermore, trauma systems are a favorable setting for de-implementation interventions as they have quality improvement teams with medical leadership, routinely collected clinical data, and performance-linked to accreditation. We aim to evaluate the effectiveness of a multifaceted intervention for reducing low-value clinical practices in acute adult trauma care.

**Methods:**

We will conduct a pragmatic cluster randomized controlled trial (cRCT) embedded in a Canadian provincial quality assurance program. Level I–III trauma centers (*n* = 30) will be randomized (1:1) to receive simple A&F (control) or a multifaceted intervention (intervention). The intervention, developed using extensive background work and *UK Medical Research Council* guidelines*,* includes an A&F report, educational meetings, and facilitation visits. The primary outcome will be the use of low-value initial diagnostic imaging, assessed at the patient level using routinely collected trauma registry data. Secondary outcomes will be low-value specialist consultation, low-value repeat imaging after a patient transfer, unintended consequences, determinants for successful implementation, and incremental cost-effectiveness ratios.

**Discussion:**

On completion of the cRCT, if the intervention is effective and cost-effective, the multifaceted intervention will be integrated into trauma systems across Canada. Medium and long-term benefits may include a reduction in adverse events for patients and an increase in resource availability. The proposed intervention targets a problem identified by stakeholders, is based on extensive background work, was developed using a partnership approach, is low-cost, and is linked to accreditation. There will be no attrition, identification, or recruitment bias as the intervention is mandatory in line with trauma center designation requirements, and all outcomes will be assessed with routinely collected data. However, investigators cannot be blinded to group allocation and there is a possibility of contamination bias that will be minimized by conducting intervention refinement only with participants in the intervention arm.

**Trial registration:**

This protocol has been registered on ClinicalTrials.gov (February 24, 2023, #NCT05744154).

**Supplementary Information:**

The online version contains supplementary material available at 10.1186/s13012-023-01279-y.

Contributions to the literature

- We will test a low-cost, pragmatic de-implementation intervention that can be adapted to other trauma systems in high-income countries.
- Our trial will advance knowledge in de-implementation science by documenting barriers, facilitators, and strategies for successful implementation.
- Reducing low-value trauma care may reduce adverse events for patients and free up healthcare resources.

## Background

Low-value clinical practices are *tests or treatments that are not supported by evidence and may cause unnecessary harm *[1]. They expose patients to adverse events such as avoidable irradiation, postoperative complications, medication or transfusion side effects, unnecessary interventions on incidental findings, and direct and indirect expenses [2–5]. They are also a major barrier to timely access to appropriate care and threaten the sustainability of modern healthcare systems [2–4]. Stakeholders in high-income countries have expressed the urgent need to develop strategies to address the burden of low-value care with interventions targeting de-implementation [6–9]. De-implementation is defined here as *discontinuing or abandoning practices that are not proven to be effective, are less effective or less cost-effective than an alternative practice, or are potentially harmful *[10, 11].

Audit & Feedback (A&F), *a summary of the clinical performance of healthcare provided over a specified period of time*, has been shown to have a modest effect on the de-implementation of low-value practices [12]. Organizations such as National Audit Commissioners advocate for enhancing A&F to support clinicians in their use of feedback data [13, 14]. Theory and evidence, largely based on the implementation of *high-value *practices, suggest that multifaceted interventions addressing determinants for success (barriers and facilitators) may be more effective and cost-effective than simple A&F [12, 15, 16]. However, the mechanisms for change, barriers, and facilitators for de-implementation differ from those for implementation [16, 17]. As such, studies on the effectiveness of interventions designed to increase *high-value* care may not be generalizable to the reduction of *low-value *practices. There is therefore a need for research on the incremental effectiveness of multifaceted interventions over simple A&F for de-implementation [18]. Additionally, although the barriers and facilitators of de-implementation have been well documented [17, 19], experts acknowledge the critical need for research on the effectiveness of interventions tailored to these determinants for success to support the de-implementation of low-value practices [17, 20–23].

Injury is the leading cause of productive life years lost and is second only to heart and stroke disease for in-patient costs [24]. Given the need to make rapid decisions in a context of multiple competing diagnostic and therapeutic options, trauma care is a high-risk setting for low-value care. Trauma quality assurance programs in place in Canada [25–29] and worldwide [30–32] have been associated with improvements in quality of care [33–35], but they are based on simple A&F and exclusively target adherence to *high-value *clinical practices. Low-value practices in trauma care are frequent, subject to significant inter-hospital variation [36, 37], and are associated with increased complications, length of stay, and in-patient costs [36]. Trauma systems are a favorable setting for de-implementation interventions as they possess many documented facilitators including quality improvement teams with medical leadership, routinely collected clinical data, and performance linked to accreditation [19]. Furthermore, potential gains are huge due to the resource-intensive nature of trauma care. Trauma systems are thus the ideal setting to advance knowledge on de-implementation of low-value care.

Our primary objective is to assess the effectiveness of a multifaceted intervention embedded in a provincial quality assurance program compared to simple A&F to reduce low-value adult acute trauma care. Secondary objectives are to (i) identify the mechanisms associated with a successful implementation of the intervention, (ii) evaluate whether intervention effectiveness changes over time, (iii) assess the effect of the intervention on clinical outcomes and resource use, (iv) assess unintended consequences, and (v) evaluate cost-effectiveness.

## Methods

This protocol is reported according to the Standard Protocol Items: Recommendations for Interventional Trials (SPIRIT) statement (Additional file 1) [38] and the Consolidated Standards of Reporting Trials (CONSORT) extension for cluster trials (Additional File 2) [39].

### Trial design

We will conduct a parallel arm, pragmatic, superiority cluster randomized trial (cRCT). The trial is pragmatic as it is embedded in a Canadian provincial trauma quality assurance program. The trial scored 5/5 on 6 of the 9 domains of the PRagmatic Explanatory Continuum Indicator Summary (PRECIS 2) tool, 3/5 for organization intervention and 4/5 for the outcome, indicating a high level of pragmatism (Additional file 3: Table S1) [40]. As the trial will be based on routinely collected data that are available at no extra cost, we will use a baseline observation period pre-randomization to increase study power [41]. We will randomize at the trauma center level because quality programs operate at the local trauma committee level in each trauma center. A stepped wedge design will not be used as the minimum 1-year rollout period is unacceptably long for stakeholders and parallel arm cRCTs have fewer risks of bias than stepped wedge cRCTs [42].

### Setting

The trial will be embedded in the *Québec Trauma Care Continuum*, a provincial regionalized trauma system comprising 57 adult trauma centers of which 3 are level I (highly specialized urban centers), 5 are level II (similar capacity to level I but in smaller cities), 22 are level III (hospitals in small towns transferring most major trauma to level I/II centers after stabilization), and 27 are level IV (rural community hospitals). All centers undergo periodic verification in line with designation, conducted by the *Institut national d’excellence en santé et services sociaux* (*INESSS*) and overseen by the Ministry of Health and Social Services [43]. Verification includes simple A&F on adherence to *high-value* care and risk-adjusted outcomes and submission of an action plan within 6 months of reception of the A&F report. Local trauma committees in each center are required to ensure the quality of the trauma program according to designation requirements. Committees include the program medical director (chair), the program manager, heads of critical care, emergency and surgical departments, and multidisciplinary services, and a hospital administrator. Quality improvement activities include trimestral committee meetings with chart review and discussions with clinical and administrative leads locally and at referring centers to identify improvement strategies such as the development of local care protocols. Formal letters of agreement are signed by heads of clinical departments to operate changes in their services when required.

### Inclusion/exclusion criteria and recruitment

We will include all adult level I–III trauma centers in the Québec *Trauma Care Continuum* (*n*= 30). All centers will be recruited as the trial will be embedded in the 2023 evaluation cycle of the provincial trauma quality assurance program, which is mandatory [43]. Level IV centers will not be included as most see fewer than 20 trauma patients per year. Patient-level inclusion criteria will be all adult (> 16 years of age) admissions with a primary diagnosis of injury to any of these trauma centers. Admissions with a primary diagnosis of thermal injuries, foreign bodies, drowning, or late effects of injuries will be excluded as diagnostic and therapeutic approaches for these diagnoses are distinct [44].

### Intervention development

The multifaceted intervention was developed using extensive background work, conducted according to recommended steps for de-implementation [16, 45, 46]. First, we identified 63 potentially low-value practices through a scoping review and clinician survey [47]. Second, we synthesized evidence on the benefits, harms, and cost-effectiveness of these practices [48–51]. Third, we measured their incidence using trauma registry data [37]. Fourth, two panels of international experts (76% participation) and local stakeholders (94% participation) and three patient–partners (75% participation) reached an evidence-informed consensus on 16 practices that should be targeted for de-implementation [52]. Fifth, we derived quality indicators for 12 of these practices with trauma registry data and showed that 6 had moderate to high validity [36]. These quality indicators target (i) initial diagnostic imaging (head, cervical spine, or whole-body computed tomography [CT] in low-risk patients), (ii) specialist consultation (neurosurgical consult for mild traumatic brain injury and spine service consult for isolated transverse process fractures), and (iii) repeat imaging for transfers (repeat scan in patients with no disease progression and no additional details needed). Based on this work, we developed a multifaceted intervention with clinical and implementation science experts, patient–partners, and accreditation professionals using *UK Medical Research Council guidelines for the Development of Complex Interventions* (Additional file 3: Figure S1) [53, 54]. We matched frequently reported barriers and facilitators reported by experts in our consensus study [52] and documented in a knowledge synthesis [19] to implementation strategies using the *Consolidated Framework for Implementation Research (CFIR)-Experts Recommendations for Implementing Change (ERIC) tool* (Additional file 3: Table S2) [45, 55, 56].

### Trial interventions

Trial interventions will be delivered by research team members twice over two evaluation cycles (12 months per cycle with a refinement phase in the first cycle; Additional file 3: Fig. S1, Table S3).

The intervention group will receive the multifaceted intervention (Additional file 3: Table S4, Figures S2.1 & S2.2), which includes (1) refinement with end users; (2) an A&F report sent to local governing authorities presenting the following: performance compared to peers (simple A&F), a summary message indicating if action is required and a list of potential actions; (3) educational materials (a clinical vignette; consequences of the practice; links to practice guidelines, clinical decision rules and shared decision-making tools; a case review tool); (4) virtual educational meetings with the local trauma medical director, trauma program manager and data analyst on how to use the report and the case review tool and how to assess their barriers and facilitators to prepare their own action plan with the CFIR-ERIC matching tool [57]; and (5) two virtual facilitation visits 2 and 4 months after the transmission of the report (or at more appropriate times depending on needs) to support committees in preparing their action plan.

**Fig. 1:**
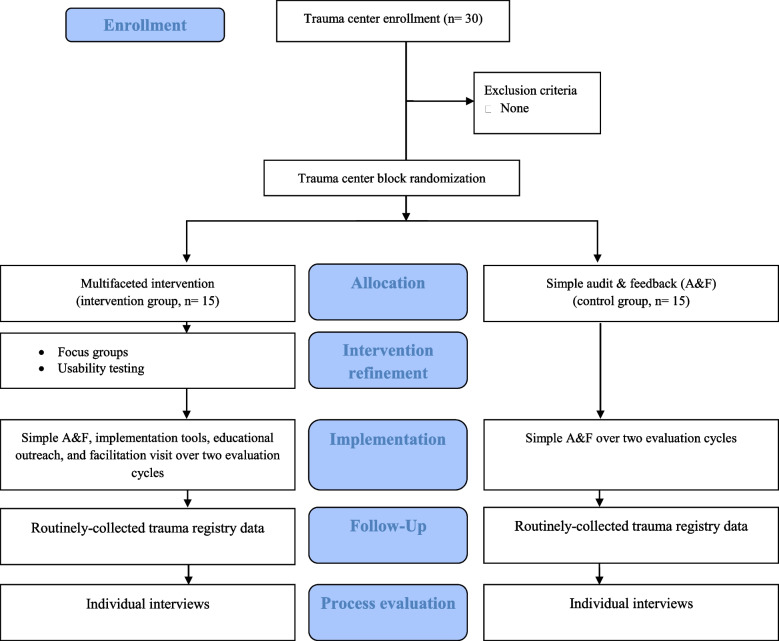
Implementation over two evaluation cycles

In line with recommendations [54] and to avoid contamination, we have embedded the refinement phase in the intervention (Additional file 3: Figure S1, Table S3, Fig. 1). We will first conduct four 60-min focus groups with 8–10 local trauma committee members [58] in trauma centers randomized to the intervention arm (stratified by designation level), with additional focus groups to obtain informational redundancy if required [59]. We will use a semi-structured interview guide (Additional file 3: Table S5) to gather reactions to the prototype, generate ideas on potential modifications, and identify other potential barriers and facilitators using the CFIR[60]. Second, we will recruit 5–8 end users [61] in the intervention arm and not involved in the previous phases to test intervention usability using a *Think Aloud Protocol *[62, 63]. We will use a one-to-one approach asking participants to articulate their reactions and their understanding of the information while going through the A&F report and the educational materials. We will modify the intervention in the presence of problems considered important by end users (e.g., difficulty in interpreting quality indicators) or in the presence of more minor problems (e.g., aesthetic details regarding the presentation of materials) reported frequently (by ≥ 5/8 end users). We will repeat this process with 5–8 additional end users until no major or frequent problems remain [61]. The intervention will be refined iteratively across focus group and usability testing rounds. We will strive for equal representation of males and females for focus groups and usability testing. Participation in this stage will also be balanced for clinical experience (i.e., < 2, 2–10, > 10 years) and disciplines (e.g., surgery, emergency medicine, radiology, critical care, nursing, management).

The control group will receive the quality improvement intervention currently in place in the *Québec Trauma Care Continuum* (i.e., simple A&F report presenting their performance compared to peers on quality indicators measuring adherence to *high-value* care and risk-adjusted outcomes) with the addition of quality indicators on low-value care (already planned for the 2023 evaluation cycle). Simple A&F was chosen for the control because it is standard practice in the *Québec Trauma Care Continuum *and in most integrated trauma systems and the effectiveness of simple A&F for de-implementation has been studied [12].

### Randomization, allocation, blinding, and adherence

Centers will be randomized to simple A&F (control) or the multifaceted intervention (intervention) in a 1:1 ratio. An independent statistician at the Ottawa Methods Center (outside of the province in which the trial will be conducted and fully independent from the sites) will generate the allocation sequence using the covariate-constrained allocation technique [64] to ensure that study arms are balanced for designation level (I/II and III), cluster size, and the pre-intervention measure of the primary outcome. The allocation sequence will only be revealed on trial initiation. An independent INESSS professional will allocate centers to their randomization group. Local trauma committees will not be told explicitly what aspect of the intervention is randomized; they will only be aware that there are two variations of the intervention. Data extractors and data analysts will be blinded to group allocation. Due to the nature of the intervention, it will not be possible to blind the investigators to allocation groups. To minimize non-adherence and contamination, only participants in the intervention arm will participate in the refinement phase. We do not envisage major problems with adherence because quality assurance activities are mandatory in line with designation requirements [43].

### Outcome measures

Our primary outcome is low-value initial diagnostic imaging, calculated as the proportion of low-risk patients who receive head, cervical spine, or whole-body CT in the emergency department (Additional file 3: Table S6). This indicator was selected on consultation with our advisory committee as it applies to the most patients, is considered to have the greatest consequences in terms of adverse effects (unnecessary radiation, delays in care, incidental findings) and resource use (availability of CT scans, staff workload, costs), and is a good indicator of global overuse in a trauma system [36]. Secondary outcomes are low-value specialist consultation (neurosurgical and spine) and repeat imaging for transfers. Other secondary outcomes are mortality, complications, resource use (intensive care unit [ICU] and hospital length of stay), and unintended consequences (unplanned readmission, missed injuries); determinants for successful implementation of the intervention (process evaluation); and incremental cost-effectiveness ratios (economic evaluation).

Follow-up will be based on continuous data collection at each site, which is mandatory in line with designation requirements. Sites will be followed up over the 18-month interval (6 to 24 months) after implementation over two evaluation cycles using these data (Fig. 1, Additional file 3: Table S3). Our advisory committee recommended evaluation over two cycles as identifying problems, implementing solutions and operating change takes place over more than one cycle [65]. We will allow for a 6-month lag period post-implementation in the first cycle, corresponding to the time allotted to centers to submit an action plan. Because data collection is mandatory in all centers, we do not anticipate any loss to follow-up.

### Study data

Data on low-value practices, mortality, complications, unplanned readmissions, ICU and hospital length of stay, and missed injuries will be extracted from the provincial trauma registry, used successfully in recent work [36]. Data collection is mandatory for all transfers and/or admissions with a primary diagnosis of injury in all provincial trauma centers [66]. Chart data are extracted in each center using coding protocols and are centralized at the Ministry of Health where they are submitted to data quality protocols. Supervision by a data manager, on-going training, an electronic forum, and quarterly meetings with stakeholders are used to ensure data quality. A recent re-abstraction of a random sample of 80 patient charts stratified by injury type and severity suggested 98% concordance on fields used for A&F (unpublished data).

### Process evaluation

We will assess fidelity, reach, and contextual factors using *Medical Research Council guidance on process evaluation of complex interventions *[67]. To assess fidelity, we will monitor the ability to deliver the multifaceted intervention and simple A&F as intended. The research team member delivering the intervention will document whether intervention components were delivered as planned and describe the challenges encountered. We will also evaluate the download of educational materials (e.g., case revision tool, clinical decision rules, shared decision-making tools) using Google Analytics [68] and examine submitted action plans to document whether they address feedback and describe strategies to facilitate de-implementation based on a local assessment of barriers and facilitators [69]. To assess reach, we will conduct 20 individual interviews (recommended sample size for informational redundancy) [70] with medical directors and trauma program managers in 20 participating centers (10 in each arm, stratified by designation level), 1 to 2 months before the end of each evaluation cycle (Fig. 1, Additional file 3: Table S3). They will be asked about strategies used to disseminate feedback to clinicians involved in trauma care, challenges in implementing the action plan, and unintended consequences of the intervention (e.g., difficulty planning transfer management without scans). We will also evaluate contextual influences on feedback responses, including elements within (e.g., competing priorities, support and commitment of decision makers, available resources) and outside the trauma center (e.g., national initiatives, policies, discussions with clinicians in other centers) [67]. Finally, at the end of the trial (months 27 to 30; Fig. 1, Additional file 3: Table S3), we will conduct 20 individual interviews (10 in each arm) with clinicians working in participating centers (stratified by designation level) to determine their recall and understanding of feedback received and the actions taken to change the practices in their organization. These clinicians will be identified through trauma program managers who will not be given any information about the purpose of the interviews to minimize selection bias. We will strive for an equal representation of males and females and of clinicians with different clinical experiences and disciplines for interviews.

### Sample size calculations

As all level I–III trauma centers will participate in this trial, we calculated the statistical power to detect a minimum important difference with a fixed number of clusters and patients per cluster (Additional file 3: Table S6). All sample size parameter values were accurately estimated by historical data from four 18-month observation periods between April 1, 2017, and March 31, 2020, using trauma registry data from the 30 level I–III trauma centers in the *Québec Trauma Care Continuum *[36]. Based on stakeholder input, a 25% relative difference in the primary outcome (proportion of patients with low-value initial diagnostic imaging) would be considered a minimum important difference. With 15 clusters in arm 1 and 15 clusters in arm 2 and an average of 285 patients per cluster period in a parallel arm cRCT with a baseline observation period, we will achieve 90% power to detect a 25% relative difference between the intervention and control arms (0.14 in the control arm versus 0.105 in the intervention arm) at a two-sided 5% level of significance [71]. We assumed a within-period intra-cluster correlation coefficient of 0.044, and a cluster autocorrelation coefficient of 0.984 (correlation from before to after). Given that the median observed absolute difference for interventions targeting de-implementation in the most recent meta-analysis on the topic was 10.5% [12], an absolute difference of 3.5% is considered plausible. For our secondary outcomes, we will have 90% power to detect absolute differences of 7.4% and 16.0% in neurosurgical consultation and repeat imaging for transfers, respectively [71].

### Data analysis plan

All statistical analyses will be based on an intention-to-treat principle and will be conducted by a statistician blinded to group assignment. Analysis of the primary outcome will be conducted at the individual patient level using weighted independence estimating equations with robust standard errors to provide valid statistical inference for the cluster-average target estimand [72]. A small sample correction will be applied to control for the small number of clusters [73]. To produce the intervention effect as a relative risk with a 95% confidence interval, we will use modified robust Poisson regression for clustered data [74]. To obtain correct type I error rates, variables used in the covariate-constrained allocation will be included as covariates (volume, designation level, and baseline proportion), and to improve power and precision [75], the analysis will adjust for prespecified patient risk factors: age, comorbidities, and injury severity (anatomical and physiological parameters). Secondary outcomes measured as binary variables will be analyzed using a similar approach. Secondary outcomes measured as continuous variables (i.e., ICU and hospital length of stay) will be analyzed using linear regression after log transformation. To address the secondary objective of evaluating trends over time, we will model repeated outcomes over 6-month intervals and include time and group-by-time interactions. We will use least square mean differences between the arms to determine whether outcomes have changed over time [76]. There are missing data on covariables in the registry, which will be handled with multiple imputation, shown to lead to accurate effect estimates for trauma quality indicators in simulations [77–79].

We will synthesize qualitative data from transcribed focus groups and individual interviews through thematic analysis [80] using QDA miner software (Provalis Research, Montréal, Canada) [81]. We chose thematic analysis because it allows for data organization and reduction, which facilitates a comprehensible description and interpretation of relevant elements [82]. To ensure credibility and reliability, peer debriefing will be carried out by experienced researchers at different analysis stages [83, 84]. We will ensure transferability using purposive sampling to maximize the chances of obtaining information that is relevant to the research objectives [83]. Credibility and trustworthiness will be reinforced by involving a coder in qualitative data analysis with no knowledge of the subject and by independent validation of the entire data analysis by a member of the research team [80]. We will use descriptive statistics to report on the sex and gender, experience, and discipline of participants and to assess intervention fidelity. To assess adherence and contamination, we will analyze process data for external influences and the use of implementation strategies in control centers. We will triangulate data on intervention effectiveness and process evaluation using a joint display to explain findings (i.e., why the intervention was effective or not) [58, 85, 86].

Effectiveness data will only be analyzed at the end of the trial. Process evaluation data collected at the end of the first cycle will be analyzed to inform intervention refinements for the second cycle, and data collected at the end of the trial will be used to refine the intervention prior to upscaling.

#### Subgroup analyses

We have planned subgroup analyses by designation level, baseline proportion of low-value initial imaging, patient sex (information on patient gender is not available), and patient age for effectiveness; and by clinician gender, age, years in practice, and discipline for the refinement of intervention and for process evaluation.

#### Sensitivity analysis

If contamination is present, we will use an instrumental variable adjustment method [87] in sensitivity analyses. To evaluate the influence of non-adherence, we will conduct per-protocol analyses. To assess the influence of multiple imputation, we will conduct complete case analyses. Fine and Grey models will be used to assess the influence of competing risks of mortality for analyses on hospital and ICU length of stay.

### Economic evaluation

We are currently conducting an early economic evaluation examining the potential benefit of the multifaceted intervention, which has informed intervention development. This includes a systematic review of the cost-effectiveness of A&F interventions [15], a budget impact analysis of low-value practices and a simulation-based early economic evaluation to estimate the potential cost-effectiveness of a hypothetical multifaceted intervention targeting low-value trauma care (initial results suggest the intervention is always dominant). Following the trial, we will update the simulation model with trial data to evaluate the cost-effectiveness of the true intervention. The economic evaluation will be conducted according to Canadian Agency Guidelines for Economic Evaluation of Health Technologies [88] and results will be reported according to the Consolidated Health Economic Evaluation Reporting Standards 2022 statement [89].

### Trial management

The main coordinating center will be at the CHU de Québec—Université Laval Research Center. Patient-level data from the trauma registry is hosted at the Ministry of Health and Social Services, and an extraction of this database with no patient-identifiable information will be accessed by the trial analyst (blinded to group allocation) through a password-protected portal at *INESSS* offices. Qualitative data from intervention refinement and process evaluations will be kept in password-protected computers in locked offices at the CHU de Québec-UL research center. Our trial coordinator will work with an *INESSS* professional to coordinate trial initiation and recruitment/randomization, implement the intervention, and obtain trauma registry data. They will also be responsible for liaising with trauma program managers in each center and coordinating the collection of qualitative data for the process evaluation. The trial steering committee, co-led by the two principal investigators (LM, MB), includes a representative of *INESSS* (CT), trauma medical directors of the three regional trauma committees (CM, TR, SS), patient partners (GP, PR) and equity, diversity, and inclusion champions (HW, NY). The committee will oversee trial conduct and knowledge translation activities and ensure we meet project deliverables according to timelines. A data monitoring committee will not be used as data for effectiveness analysis is not collected within the trial and the risk to participants is considered very low. Any trial modifications will be submitted to the trial steering committee for approval, recorded in our registered protocol, and reported in final manuscripts.

### Stakeholder, patient, and public involvement

Our primary stakeholder partner is the provincial paragovernmental organization responsible for overseeing healthcare quality (INESSS). Our research team has been working in collaboration with this organization since 2010. They have been involved in designing the trial so it can be embedded in their 2023 evaluation cycle, and they will be involved in all phases of trial conduct. The trial has also been designed in collaboration with the *Trauma Association of Canada* (association of clinicians involved in trauma care across Canada) and *Health Services Organisation* (responsible for hospital accreditation programs in Canada). The design of this cRCT has also been informed by national research collaboratives, the *Canadian Traumatic brain injury Research Consortium* and the *Canadian Critical Care Trials Group*, and national knowledge translation networks, *Choosing Wisely Canada*, and the *A&F Metalab*. To ensure trial results are relevant to other high-income countries with similar trauma care infrastructures, we have partnered with the UK *Trauma Audit Research Network* and *Victoria State Trauma Outcomes Registry and Monitoring group*in Australia. Three patients (PR, GP, MA) with varying injury profiles were integrated in the consensus process to select low-value practices targeted in this trial [52]. We first conducted a preparatory meeting to explore their perceptions on low-value trauma care, establish their priorities, and prepare for their integration. Patient perceptions and priorities were presented by a patient at the consensus meeting. All patients participated in the meeting and voted on practices to be retained. Patients will not be involved in intervention refinement as they are not end users [90]. However, two patients (PR, GP) are on the trial steering committee. No members of the general public are involved.

### Ethics and dissemination

This trial has been approved by the CHU-de-Québec – Université Laval research ethics board (#113,664). Our dissemination plan was designed using published guidelines [91] and includes (i) one-page visual summaries on partner organization websites, (ii) peer-reviewed publications, (iii) presentations at clinical and academic (de/implementation science) conferences and to end users (patient support groups, clinicians, accreditation authorities), (iv) media channels and policy briefs, and (v) half-day workshop with implementation science and accreditation stakeholders on the development multifaceted interventions targeting de-implementation.

## Discussion

The intervention has a high probability of success because it targets a problem identified by stakeholders, is based on extensive background work, will be refined with end users considering barriers and facilitators, is low-cost (embedded in an existing quality assurance platform, based on routinely collected data), and is linked to accreditation. This setting provides a unique opportunity to develop a cost-efficient, acceptable, sustainable intervention that can be integrated into existing trauma quality assurance platforms. Bias is anticipated to be minimal (no attrition, identification, or recruitment bias). However, there may be differences in engagement that will be explored through process evaluation. Furthermore, investigators cannot be blinded to group allocation and there is a possibility of contamination bias that will be minimized by conducting intervention refinement only with participants in the intervention arm and will be assessed in process evaluations.

This project will advance de-implementation science through the application of knowledge on barriers and facilitators to intervention design and on the incremental effectiveness of a multifaceted de-implementation intervention over simple A&F in a pragmatic setting. If the intervention is effective and cost-effective, we will upscale across Canada with national and provincial trauma authorities and promote uptake in other healthcare domains. We will also explore international uptake through collaborating organizations in the UK, Australia, and the USA. This intervention has the potential to reduce the adverse effects and indirect expenses of low-value trauma care for patients and families. It could also free up resources, reduce delays to care, and reduce clinician workload, ultimately improving efficiency at a time of unprecedented strain on healthcare resources.

## Supplementary Information

**Additional file 1.** SPIRIT 2013 Checklist: Recommended items to address in a clinical trial protocol and related documents*.

**Additional file 2.** CONSORT 2010 checklist of information to include when reporting a cluster randomised trial.

**Additional file 3:** **Table S1.** PRECIS-2 scores for trial domains with rationale. **Figure S1.** Integration of the project within UK Medical Research Council guidelines for the Development of Complex Interventions. **Table S2.** Matching of barriers with implementation strategies according to the CFIR-ERIC tool. **Table S3.** Schedule of enrolment, interventions, and assessments. **Table 4.** Intervention prototype as per the TIDieR checklist. **Figure S2.1.** Example of a page of the A&F report for one quality indicator (intervention arm). **Figure S2.2.** Example of output from patient chart revision tool† (intervention arm). **Table S5.** Questions for the semi-structured interviews for focus groups (intervention refinement). **Table S6.** Sample size calculation for primary and selected secondary outcomes.

## Data Availability

Individual patient data are held by provincial authorities and cannot be transmitted by study investigators. Supporting material will be made available through the research program website. Data from intervention refinement and process evaluations will be made available on request.

## Abbreviations

A&F: Audit & feedback
CFIR-ERIC: Consolidated Framework for Implementation Research-Experts Recommendations for Implementing Change
CHU: Centre Hospitalier Universitaire
CONSORT: Consolidated Standards of Reporting Trials
cRCT: Cluster randomized controlled trial
CT: Computed tomography
ICU: Intensive care unit
INESSS: Institut d’Excellence en Santé et en Services Sociaux
PRECIS-2: Pragmatic Explanatory Continuum Indicator Summary
QDA: Qualitative data analysis
SPIRIT: Standard Protocol Items: Recommendations for Interventional Trials
