# Supplementary material for: Evaluating the effectiveness of a multifaceted intervention to reduce low-value care in adults hospitalized following trauma: a protocol for a pragmatic cluster randomized controlled trial
Source: Implement Sci. 2023 Jul 7;18:27. doi: 10.1186/s13012-023-01279-y (PMC10329386; doi:10.1186/s13012-023-01279-y)
Supplement: Supplementary file 3 — Additional file 3: Table S1. PRECIS-2 scores for trial domains with rationale. Figure S1. Integration of the project within UK Medical Research Council guidelines for the Development of Complex Interventions. Table S2. Matching of barriers with implementation strategies according to the CFIR-ERIC tool. Table S3. Schedule of enrolment, interventions, and assessments. Table 4. Intervention prototype as per the TIDieR checklist. Figure S2.1. Example of a page of the A&F report for one quality indicator (intervention arm). Figure S2.2. Example of output from patient chart revision tool† (intervention arm). Table S5. Questions for the semi-structured interviews for focus groups (intervention refinement). Table S6. Sample size calculation for primary and selected secondary outcomes. [file 13012_2023_1279_MOESM3_ESM.docx]

**Supplementary Table 1. PRECIS-2 scores for trial domains with rationale**

|  | Domain | Score | Rationale |
| --- | --- | --- | --- |
| 1 | Eligibility Criteria | 5 | Identical to those receiving usual care (all centers are eligible) |
| 2 | Recruitment Path | 5 | Same process to recruit as in usual care (all centers will be recruited) |
| 3 | Setting | 5 | Identical to usual care setting |
| 4 | Organisation intervention | 3 | Intervention arm will include educational materials and facilitation visits which will require additional resources to usual care (simple audit & feedback) |
| 5 | Flex of experimental intervention – Delivery | 5 | Identical flexibility to usual care (centers are required by provincial authorities to submit action plan within 6 months) |
| 6 | Flex of experimental intervention – Adherence | 5 | Identical adherence to usual care (mandatory in line with designation requirements) |
| 7 | Follow up | 5 | Intensity of measurement and follow up identical to usual care – routine data collection in all centers |
| 8 | Outcome | 4 | Participants involved in selection of outcome through an evidence-based local stakeholder consensus process |
| 9 | Analysis | 5 | All data are included; intention-to-treat approach used |

**Supplementary Figure 1. Integration of the project within *UK Medical Research Council guidelines for the Development of Complex Interventions***

**
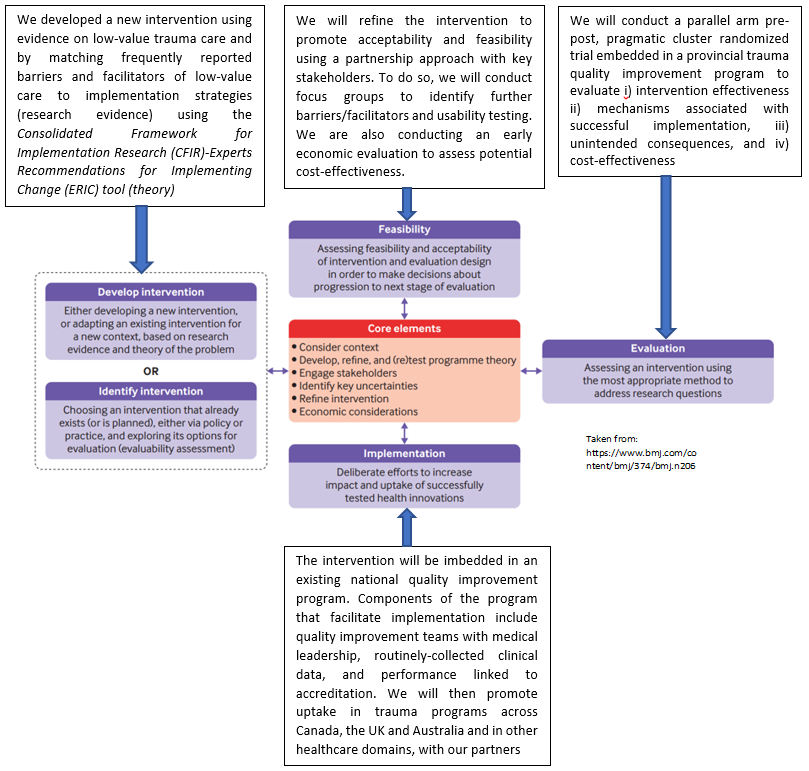
**

**Supplementary Table 2. Matching of barriers with implementation strategies according to the CFIR-ERIC tool**

Waltz TJ, Powell. BJ, Fernandez ME, Abadie B, Damschroder LJ (2019). Choosing implementation strategies to address contextual barriers: diversity in recommendations and future directions. *Implementation Science*, 14:42. https://cfirguide.org/choosing-strategies/

| **Barriers identified in the literature and previous consensus study** | **Type of barriers according to the CFIR** | **Most relevant implementation strategy according to the CFIR-ERIC matching tool** |
| --- | --- | --- |
| Beliefs and opinions of providers on low value practices | - Relative advantage - Culture - Knowledge and beliefs about the intervention | - Identify and prepare champion - Conduct educational meeting |
| Fear of medical error by de-implementing low value practice | - Relative advantage - Tension for change - Compatibility | - Identify and prepare champion - Conduct local consensus discussion - Promote adaptability |
| Defensive attitude (better to provide the low value practice in the presence of doubt) | - Relative advantage - Culture - Tension for change - Compatibility | - Identify and prepare champion - Conduct local consensus discussion |
| Desire to meet patients’ expectations | - Patient needs and resources - Patients/Customers | - Obtain and use patients/consumers and family feedback - Involve patients/consumers and family members - Conduct local needs assessment - Provide shared decision-making tools |
| Motivation and commitment to restrict unnecessary care | - Goals and feedback - Self-efficacy - Individual stage of change | - Audit & provide feedback - Develop a formal implementation blueprint - Provide ongoing consultation - Conduct ongoing training - Make training dynamic |
| Provider knowledge about low value practices | - Access to knowledge and information - Knowledge and belief about the intervention | - Conduct educational meeting - Develop and distribute educational material |
| Awareness of an agreement with guidelines | - Access to knowledge and information - Knowledge and belief about the intervention | - Conduct educational meeting - Develop and distribute educational material - Provide clinical decision rules |
| Sense of ownership and participation in the project | - Learning climate - Leadership engagement - Opinion leaders - Formally appointed internal implementation leaders - Champions | - Facilitation - Involve executive boards - Identify and prepare champion - Inform local opinion leader |
| Lack of database to monitor the quality of care and progress of de-implementation | - Goals and feedback - Reflecting and evaluating | - Audit & provide feedback - Develop and implement tools for quality monitoring |
| Having the right information from database | - Goals and feedback - Reflecting and evaluating | - Audit & provide feedback - Develop and implement tools for quality monitoring |
| Lack of time | - Complexity - Available resources | - Develop a formal implementation blueprint - Promote adaptability - Access new funding |
| Lack of tools to facilitate change in practice | - Complexity - Available resources - Self-efficacy - Planning | - Develop a formal implementation blueprint - Promote adaptability - Access new funding - Provide ongoing consultation - Conduct ongoing training - Make training dynamic - Conduct local needs assessment |
| Change required in the existing workflow or referral patterns | - Complexity - Compatibility - Learning climate - Self-efficacy - Planning | - Develop a formal implementation blueprint - Promote adaptability - Facilitation - Provide ongoing consultation - Conduct ongoing training - Make training dynamic - Conduct local needs assessment - Assess and redesign workflow |
| Economic political context financial incentives to do less | - External policy & incentives | - Involve executive board - Alter incentive/allowance structure |

**Definitions**

Perry CK, Damschroder LJ, Hemler JR, Woodson TT, Ono SS, Cohen DJ (2019). Specifying and comparing implementation strategies across seven large. Implementation interventions: a practical application of theory. *Implementation Science,*14:32.

Powell BJ, Waltz TJ, Chinman MJ, Damschroder LJ, Smith JL, Matthieu MM, Proctor EK, Kirchner JE (2015). A refined compilation of implementation strategies: results from the Expert Recommendations for Implementing Change (ERIC) project. *Implementation Science*, 10:21.

**Access new funding:** Access new or existing money to facilitate the implementation

**Alter incentive/allowance structures:** Work to incentivize the adoption and implementation of the clinical innovation

**Assess and redesign workflow:** Observe and map current work processes and plan for desired work processes, identifying changes necessary to accommodate, encourage, or incentivize use of the clinical innovation as designed

**Audit & provide feedback**: Develop summaries of clinical performance over a specific time period, often including a comparator, and give it to clinicians and/or administrators. Summary content (e.g., nature of the data, choice of comparator) and their delivery (e.g., mode, format) are designed to modify specifically targeted behavior(s) or actions of individual practitioners, teams, or health care organizations

**Conduct educational meeting:** Hold meetings targeted toward educating multiple stakeholder groups (i.e., providers, administrators, other organizational stakeholders, community members, patients/consumers, families) about the clinical innovation and/or its implementation.

**Conduct local needs assessment:** Collect and analyze data related to the need for the innovation

**Conduct local consensus discussion:** Include providers and other stakeholders in discussions that address whether the chosen problem is important and whether the clinical innovation to address it is appropriate.

**Conduct ongoing training:** Plan for and conduct training in the clinical innovation in an ongoing way for all individuals involved with implementation and users of the clinical innovation e.g., clinicians, implementation staff, practice facilitators

**Develop a formal implementation blueprint:** Develop summaries of clinical performance over a specific time period, often including a comparator, and give it to clinicians and/or administrators. Summary content (e.g., nature of the data, choice of comparator) and their delivery (e.g., mode, format) are designed to modify specifically targeted behavior(s) or actions of individual practitioners, teams, or health care organizations

**Develop and distribute educational material:** Develop and format manuals, toolkits, and other supporting materials to make it easier for stakeholders to learn about the innovation and for clinicians to learn how to deliver the clinical innovation. This can include technology-delivered (e.g., online/smartphone-based static or

dynamic) content and health messaging. Distribute educational materials (including guidelines, manuals, and toolkits) in person, by mail, and/or electronically.

**Develop and implement tools for quality monitoring:** Develop, test, and introduce into quality-monitoring systems the right input—the appropriate language, protocols, algorithms, standards, and measures (of processes, patient/consumer outcomes, and implementation outcomes) that are often specific to the innovation being implemented

**Facilitation:** Multi-faceted interactive process of problem solving, enabling and supporting individuals, groups and organizations in their efforts to adopt and incorporate innovations into routine practices that occurs in a context of a recognized need for improvement and a supportive interpersonal relationship

**Identify and prepare champion**: Cultivate relationships with people who will champion the clinical innovation and spread the word of the need for it. This strategy includes preparing individuals for their role as champions. Champions can be internal or external to the organization.

**Inform local opinion leader:** Inform providers identified by colleagues as opinion leaders or “educationally influential” about the clinical innovation in the hopes that they will influence colleagues to adopt it

**Involve executive board:** Involve existing governing structures (e.g., boards of directors, medical staff boards of governance) in the implementation effort, including the review of data on implementation processes

**Involve patients/consumers and family members:** Engage or include patients/consumers and families in the implementation effort

**Make training dynamic:** Vary the information delivery methods to cater to different learning styles and work contexts, and shape the training in the innovation to be interactive

**Obtain and use patients/consumers and family feedback:** Develop strategies to increase patient/consumer and family feedback on the implementation effort

**Promote adaptability:** Identify the ways a clinical innovation can be tailored to meet local needs and clarify which elements of the innovation must be maintained to preserve fidelity

**Provide ongoing consultation:** Provide ongoing consultation with one or more experts in the strategies used to support implementing the innovation

**Supplementary Table 3. Schedule of enrolment, interventions, and assessments**

|  | **STUDY PERIOD** | | | | | |
| --- | --- | --- | --- | --- | --- | --- |
|  | **Enrolment** | **Allocation** | **Post-allocation** | | | **Close-out** |
| **TIMEPOINT** | ***-2m*** | **0m** | ***0-3m*** | ***3-9m*** | ***9-27m*** | ***27-30m*** |
| **ENROLMENT:** |  |  |  |  |  |  |
| **Eligibility screen** | X |  |  |  |  |  |
| **Allocation** |  | X |  |  |  |  |
| **Intervention refinement** |  |  | X |  |  |  |
|  |  |  |  |  |  |  |
| **INTERVENTIONS^1^:** |  |  |  |  |  |  |
| ***Multifaceted intervention (I)*** |  |  |  |  |  |  |
| ***Simple audit & feedback (C)*** |  |  |  |  |  |  |
|  |  |  |  |  |  |  |
| **ASSESSMENTS:** |  |  |  |  |  |  |
| ***Baseline^2^*** | X |  |  |  |  |  |
| ***Lag period*** |  |  |  | X |  |  |
| ***Effectiveness assessment*** |  |  |  |  | X |  |
| ***Process evaluation^3^*** |  |  |  |  | X | X |

^1^ Implemented twice over two 18-month evaluation cycles

^2^ Baseline observation period 18 months pre-allocation

^3^ One to two months before the end of each evaluation cycle and at the end of the trial

**Supplementary Table 4. Intervention prototype as per the TIDieR checklist**

| **Implementation strategies (what)** | **How it will be delivered (by whom to whom)** | **Timing (when)** | **Duration (how much)** | **Fidelity assessment (how well)** |
| --- | --- | --- | --- | --- |
| Conduct local needs assessment regarding the intervention and prepare champions:   - Participation of local trauma committee members and other key end users in the refinement of intervention prototype (focus groups and usability testing) | By: Co-principal applicant (Mélanie Bérubé) and research coordinator with experience in trauma care  To: Members of local trauma committees, trauma surgeons, neurosurgeons, orthopaedic surgeons, emergency physicians, radiologists, critical care physicians | 0-3 months following randomization | Four 60-minute focus groups with 8-10 local trauma committee members  5-8 individual meetings with end user for usability testing | NA |
| Involve governing structures:   - Involve the accreditation authority (INESSS) in the production and distribution of audit & feedback (A&F) reports - Send A&F report to trauma centre Chief Executive Officers (CEOs) - Send A&F report to trauma medical director and trauma program manager | By: Research team,  INESSS  To: CEOs, trauma medical director, trauma program manager | During the production of the A&F (INESSS)  Beginning of each evaluation cycle | NA | - Report consultation by CEO, medical trauma directors and trauma program coordinators |
| Audit & provide feedback: ^b^   - Performance compared to peers (simple A&F)^b^ - Performance over time^c^ - Summary message indicating if action is required | By: Research team,  INESSS  To: CEOs, trauma medical director, trauma program manager | Beginning of each evaluation cycle (3 and 15 months following randomization) | NA | - Report consultation by CEO, medical trauma directors and trauma program coordinators - Questions from CEO, medical trauma directors and trauma program coordinators regarding the report content |
| Provide educational material to prepare champions:   - Using the patient chart revision tool (see 5.2) - Preliminary list of suggested actions based on matching of barriers with implementation strategies described in Appendix 3 (e.g., inform local opinion leaders, conduct local needs assessment through chart reviews, involve patients and family members to promote clinician-patient/family making, access and redesign workflow, conduct consensus discussion with key stakeholders on required actions) - Clinical vignettes on low value practices - Consequences of low-value care - Links to supporting materials (clinical practice guidelines, clinical decision rules, clinician-patient/family shared decision- making tools) | By: Research team  INESSS  To: Members of local trauma committees | Beginning of the first evaluation cycle (month 3) | NA | - Use of the chart revision tool - Uploading of the educational materials by medical trauma directors and trauma program coordinators - Questions from medical trauma directors and trauma program coordinators regarding the educational material |
| Virtual educational meeting:   - Present quality indicators, their rationale and how to interpret results - Training on how to evaluate barriers and facilitators and select solutions with committee members using the CFIR-ERIC matching tool | By: Co-principal applicant (Mélanie Bérubé) and research coordinator with experience in trauma care  To: Medical Director, trauma program manager and data analyst | Beginning of the first evaluation cycle (month 3), within 2 to 6 weeks following the transmission of the A&F report^d^ | 60-90 minutes | - Delivery of educational components as planned - Examination of action plan to determine whether they address the feedback provided and describe the strategies suggested to facilitate deimplementation of low value practices based on a local assessment of barriers and facilitators |
| Virtual facilitation visits-Ongoing consultation:   - Interpretation of feedback - Identification of barriers and facilitators to establish their action plan - Provide support on how to tailor their action plan to meet local needs while addressing the most important issues (promote adaptability). | By: Co-principal applicant (Mélanie Bérubé) and research coordinator with experience in trauma care  To: Trauma medical director, trauma program manager | 2 and 4 months^e^ after transmission of the A&F report or at other more appropriate times depending on the needs of trauma teams^d^ | 30-60 minutes | - Delivery of facilitation visits components as planned - Examination of action plan |

^a^See below for an example

^b^Simple A&F (control intervention)

^c^For the second evaluation cycle

^d^The same components could be applied to both evaluation cycles depending on staff turnover and according to local needs.

^e^The 2- and 4-month timeline was established based on our previous experience with the implementation of high-value care based on audit & feedback report

**Supplementary Figure 2.1. Example of a page of the A&F report for one quality indicator (intervention arm)**


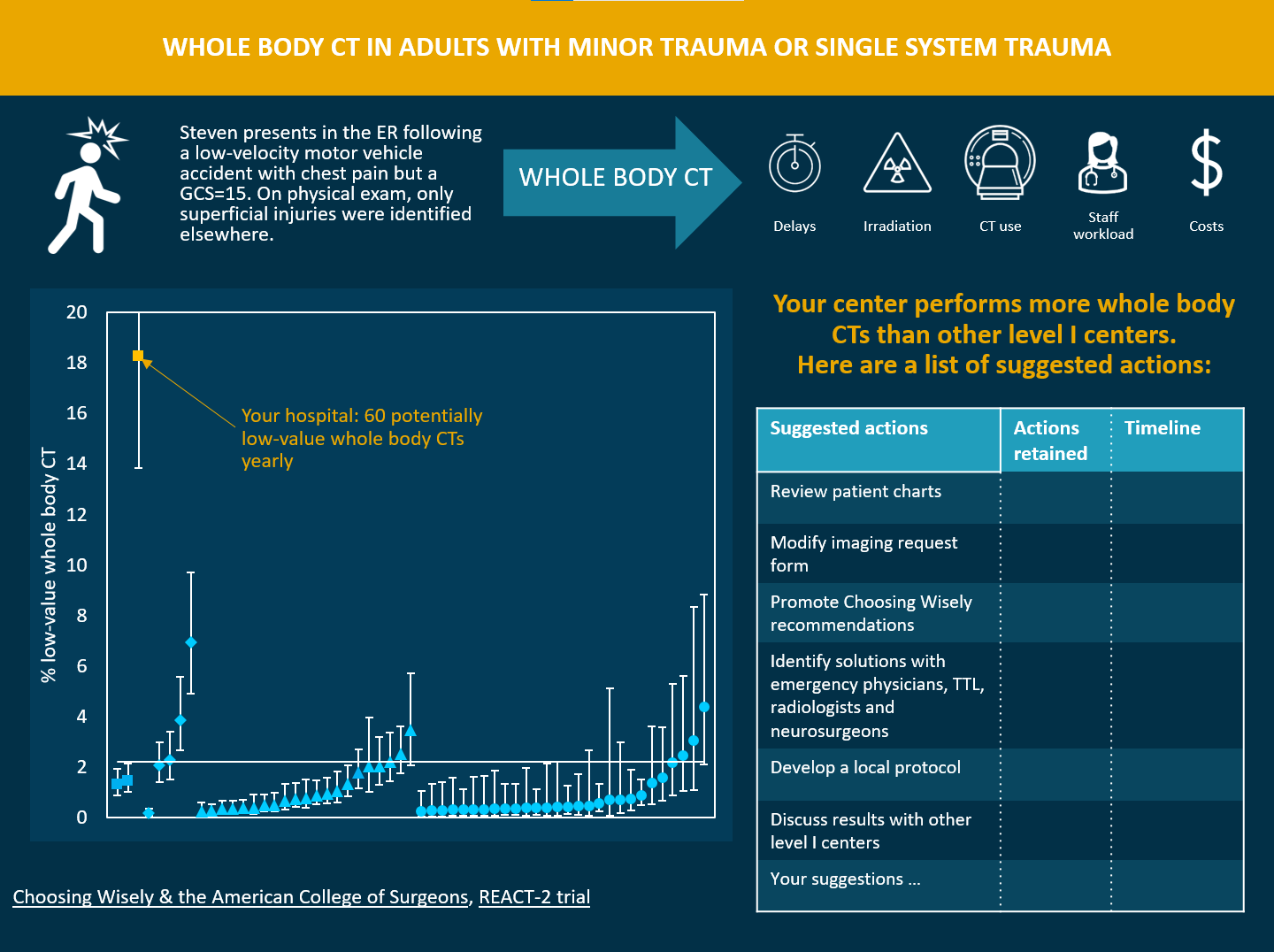


Links to supporting materials (e.g. clinical decision rules, shared decision- making tools)

Summary message indicating if action is required

Consequences of the practice

Preliminary list of suggested actions (Appendix 3)

Performance compared to peers

Clinical vignette

**Supplementary Figure 2.2. Example of output from patient chart revision tool† (intervention arm)**


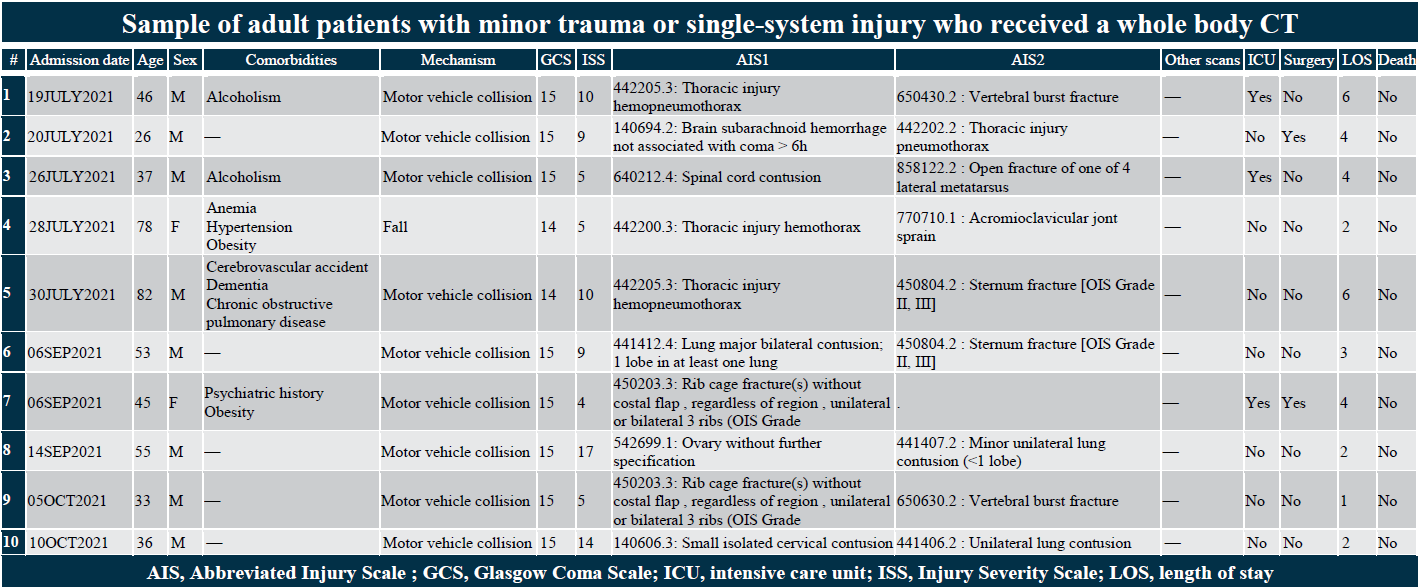


†Do not represent real cases; lists will be generated automatically from the local trauma registry interface in each center

**Supplementary Table 5. Questions for the semi-structured interviews for focus groups (intervention refinement)**

**Questionnaire**

***Assessment of barriers and facilitators***

***Low-value practices in trauma care***

1. What do you think in general of the intervention?
2. What types of changes or modifications do you think we should make to the intervention to facilitate its integration into your setting?
3. Are there any components of the intervention that should not be changed? If so, which ones?
4. What is your perception of the quality of support materials and the presentation of the intervention to facilitate its implementation? What types of information and materials regarding the intervention could be provided for individuals at your facility?
5. What do you think are the potential benefits of the intervention for the patients provided for by your organization? For instance, do you believe that the implementation of the intervention will result in better access to services, a reduction in waiting time, a decrease in adverse events?
6. What are your thoughts on current practices related to the intervention? How will the intervention address current gaps in practice?
7. What kinds of changes will be needed to implement the intervention (Changes in scope of practice? Changes in official policies? Changes in information systems or electronic record systems? Others?). Can you describe the processes that will be required to make these changes?
8. How do you think your organization’s culture (general beliefs, values that people hold about wasting resources and costs) will affect the implementation of the intervention?
9. How confident are you that you will be able to successfully implement the intervention and what gives you that level of confidence (or lack thereof)?

**Supplementary Table 6. Sample size calculation for primary and selected secondary outcomes**

| **Composite outcome** | **Low-value practice** | **Centers** | **Baseline proportion (a/n)^a^** | **wpICC** | **CAC** | **Cluster-period size** | **Detectable difference^b^** |
| --- | --- | --- | --- | --- | --- | --- | --- |
| **Primary** | | | | | | | |
| **Initial diagnostic imaging** | Head CT | 30 | 14%  (1163/8310) | 0.044 | 0.984 | 285 | 3.50% |
|  | Cervical spine CT |  |  |  |  |  |  |
|  | Whole body CT |  |  |  |  |  |  |
| **Secondary** | | | | | | | |
| **Specialist consultation^c^** | Neurosurgical consultation | 30 | 20%  (607/3069) | 0.045 | 0.836 | 106 | 7.40% |
|  | Spine consultation |  |  |  |  |  |  |
| **Repeat imaging for transfers^d^** | Repeat CT in patients with no disease progression and no additional details needed | 30 | 43%  (608/1405) | 0.186 | 0.995 | 21 | 16.00% |

CAC, Cluster Autocorrelation Coefficient; CT, computer tomography; wpICC, within-period Intraclass Cluster Coefficient

^a^ Based on average 18-month proportion over 4 observation periods between April 1^st^, 2017 and March 31^st^, 2020 using trauma registry data from the *Quebec Trauma Care Continuum*

^b^ Detectable absolute difference with 90% power

^c^ Includes consult requested in level I/II emergency department and transfer to level I/II center for consult requested by level III center

^d^ Includes CT I/II centers post-transfer and CT in level III centers for patients with a clear indication for transfer
